# Importance of Baseline Specification in Evaluating Conservation Interventions and Achieving No Net Loss of Biodiversity

**DOI:** 10.1111/cobi.12243

**Published:** 2014-02-13

**Authors:** J W Bull, A Gordon, E A Law, K B Suttle, E J Milner-Gulland

**Affiliations:** * Department of Life Sciences, Imperial College London, Silwood Park CampusAscot SL5 7PY, United Kingdom; † School of Global, Urban and Social Studies, RMIT UniversityMelbourne, Victoria 3001, Australia; ‡ Environmental Decisions Group, School of Biological Sciences, University of QueenslandBrisbane, Queensland 4072, Australia; § Department of Life Sciences & Grantham Institute for Climate Change, Imperial College London, Silwood Park CampusAscot SL5 7PY, United Kingdom

**Keywords:** biodiversity offsets, conservation planning, counterfactuals, environmental trends, frame of reference, simulation modeling, ajuste de biodiversidad, contrafactuales, marco de referencia, modelo de simulación, planificación de la conservación, tendencias ambientales

## Abstract

There is an urgent need to improve the evaluation of conservation interventions. This requires specifying an objective and a frame of reference from which to measure performance. Reference frames can be baselines (i.e., known biodiversity at a fixed point in history) or counterfactuals (i.e., a scenario that would have occurred without the intervention). Biodiversity offsets are interventions with the objective of no net loss of biodiversity (NNL). We used biodiversity offsets to analyze the effects of the choice of reference frame on whether interventions met stated objectives. We developed 2 models to investigate the implications of setting different frames of reference in regions subject to various biodiversity trends and anthropogenic impacts. First, a general analytic model evaluated offsets against a range of baseline and counterfactual specifications. Second, a simulation model then replicated these results with a complex real world case study: native grassland offsets in Melbourne, Australia. Both models showed that achieving NNL depended upon the interaction between reference frame and background biodiversity trends. With a baseline, offsets were less likely to achieve NNL where biodiversity was decreasing than where biodiversity was stable or increasing. With a no-development counterfactual, however, NNL was achievable only where biodiversity was declining. Otherwise, preventing development was better for biodiversity. Uncertainty about compliance was a stronger determinant of success than uncertainty in underlying biodiversity trends. When only development and offset locations were considered, offsets sometimes resulted in NNL, but not across an entire region. Choice of reference frame determined feasibility and effort required to attain objectives when designing and evaluating biodiversity offset schemes. We argue the choice is thus of fundamental importance for conservation policy. Our results shed light on situations in which biodiversity offsets may be an inappropriate policy instrument Importancia de la Especificación de Línea de Base en la Evaluación de Intervenciones de Conservación y la Obtención de Ninguna Pérdida Neta de la Biodiversidad

## Introduction

When setting objectives for conservation activities, or judging their efficacy after implementation, an appropriate frame of reference against which evaluation is made should be specified. This would mean considering the eventual state of the region in which conservation activities have taken place and comparing it with some known historical state (a baseline) or against some alternative scenario that would have taken place without the intervention (a counterfactual). That appropriate frames of reference are not widely used in practice is a problem for contemporary conservation (Ferraro & Pattanayak 2006; McDonald-Madden et al. 2009). Often, if a region is subject to some conservation intervention, then subsequent ecological recovery is treated as a success and further deterioration as failure. Consequently, interventions are implicitly evaluated against a baseline consisting of the fixed point in time at which intervention began, if at all. Yet a fixed baseline may be an insufficient basis for judging the true impact of interventions because ecosystems are dynamic (Nicholson et al. 2009) and can change without intervention (Ferraro & Pattanayak 2006).

The actual success or failure of an intervention depends on the frame of reference chosen to assess it against. For instance, if a region's biodiversity is decreasing in some way that conservation seeks to address, then slowing that deterioration may represent a positive outcome, even if deterioration continues in absolute terms. Likewise, conservation activity imposed on a region that is recovering from some decrease in biodiversity may be misjudged as having succeeded, even if the overall trend for recovery was not actually altered. For example, tropical forests are protected to prevent deforestation, and an expansion in protected area may be considered a success for conservation. But their effectiveness depends on how much forest would have disappeared in the absence of protection (i.e., the counterfactual scenario), which is often ignored (McDonald-Madden et al. 2009). Protected areas are instead often evaluated based on the amount of forest left standing (Adnam et al. 2008). Performance evaluation of conservation interventions should involve clearly specified counterfactuals that incorporate consideration of the likely trajectory of the target region without the intervention.

In addition to evaluation of outcomes, the choice of reference frame can shape how an intervention is structured and implemented. For example, 74% of the Aral Sea by area has changed from saline lake to semiarid scrubland over recent decades (Micklin 2007). In this case, the difference between a 1960 and a 2000 baseline as a frame of reference for conservation activities is important. So the choice of reference frame is a critical component of the process of conservation, even if it is often unstated and implicit only in conservation policy.

Reference frames are commonly applied in policy relating to climate change. Under the Kyoto Protocol, participating countries agreed to reductions in the annual percentage of greenhouse gas emissions relative to 1990 levels (UN 1998). Thus, emissions of greenhouse gases in the year 1990 are a baseline against which change is evaluated. A counterfactual could also be used; for example, actual annual emissions could be compared with calculated annual business-as-usual emissions (i.e., had the protocol never been implemented and the status quo maintained). Climate change policy also highlights the difficulties in specifying reference frames. For instance, the baseline could be adjusted to account for industrialization, incorporating a development adjustment factor to allow higher emissions for poorer countries so as not to limit socioeconomic development (Angelson 2008). The inclusion of such a factor would place a greater burden on industrialized nations to reduce emissions and increase the effort required to successfully implement climate policy. Clearly, choosing between reference frames may be controversial.

In conservation, the relationship between reference frames and outcomes has been implicitly discussed since the introduction of the concept of shifting baseline syndrome in the 1990s (Pauly 1995). The conjecture is that each successive generation of conservationists sets extant biodiversity during their early years as their own personal baseline for what is natural; thus, they mask longer term biodiversity decline. The topic of appropriate baselines also arises in restoration and rewilding literature (Alagona et al. 2012), but altogether it receives less attention in conservation than in climate policy. Even in newly developing conservation approaches, such as biodiversity offsetting, baselines can be overlooked (Quétier & Lavorel 2012; Bull et al. 2013). Given a specified frame of reference, however, scientists can tell us whether achieving a stated conservation objective is feasible and what might influence outcomes. This is what we explored.

### Defining a Frame of Reference

We use the term *reference frame* to represent a state against which conservation interventions can be evaluated through some measure of biodiversity, of which baselines and counterfactuals are both types. The choice of reference frame is ultimately a value judgment, yet there are criteria that should guide reference frame specification and practical interpretation. A useful reference frame must include at least 2 facets of environmental change in the focal region: ongoing trends in biodiversity (i.e., biodiversity trajectory) and human impacts upon biodiversity (i.e., anthropogenic impacts) (Table1).

**Table 1:** A description of the contexts for biodiversity-loss offset interventions in a dynamic socioecological system

Context	Applies to	Example options	Explanation
Anthropogenic impacts + conservation intervention	anthropogenic pressure upon target ecosystem	no development	no development, no offsets
		development only	development has negative impacts upon biodiversity, but no offsets implemented
		development with offsets	development has negative impacts upon biodiversity, offsets implemented to compensate
Biodiversity trajectory	likely biodiversity trend in the absence of anthropogenic pressure	decreasing	biodiversity would deteriorate with time, e.g., invasive weeds displacing existing species
		stable	biodiversity would remain stable (e.g., a climax habitat type)
		increasing	biodiversity would improve with time (e.g., previously exploited species increasing in abundance)

The biodiversity trajectory is the trend in biodiversity within some defined region in the hypothetical absence of any further anthropogenic development or conservation activity (including drivers caused by past anthropogenic activities or processes, such as climate change or invasive species). For instance, remnant native grasslands in Victoria, Australia, would continue to decline in conservation value in the absence of subsequent human activity, driven partly by generalist invasive species. Therefore, protection from urban development—a current anthropogenic threat—is an inadequate conservation strategy for these remnants; they must be actively managed for conservation of native species (Gordon et al. 2011).

Anthropogenic impacts are the effects of human activities taking place within the region that affect biodiversity, positively or negatively. These include negative impacts upon biodiversity from development and land use change and existing projects to safeguard biodiversity that are not part of the conservation intervention to be evaluated.

The biodiversity trajectory and existing anthropogenic impacts provide necessary background for new conservation interventions. They determine whether a baseline or counterfactual is the most appropriate frame of reference and are used to develop robust counterfactuals. Three other elements of a frame of reference must be specified: spatial scale, temporal scale, and time lags between losses and gains in biodiversity.

Frames of reference can be set at the scale of actual intervention projects (e.g., patches of vegetation directly manipulated) or of the larger landscape (e.g., manipulated patches plus surrounding areas in which interventions are not directly undertaken). Either scale may be reasonable dependent upon the situation. For example, EU agri-environment schemes (Whittingham 2007) can be evaluated by aggregating information from only individual farms involved or from the entire landscape, including areas not part of the scheme. The evaluation of the scheme's success may be scale dependent: biodiversity may increase on individual farms (i.e., at the project scale), whereas regional (i.e., landscape scale) changes in biodiversity may be negligible (Whittingham 2007).

The reference frame can be fixed at some baseline point of initial measurement, such as the outset of the intervention, or based on predicted counterfactual trends through time. These can be thought of as fixed and relative frames of reference, respectively. The biodiversity baseline in the region at the time the intervention began is often the frame of reference specified or implied for conservation initiatives. Conversely, counterfactuals may include the biodiversity trajectory for the region had there been no development or intervention (i.e., the status quo or no-development scenario) or the worst-case scenario of development without compensatory conservation (i.e., development only). While both types of reference frame are subject to uncertainties, counterfactuals are inherently more uncertain because they involve predicting future trends in addition to taking measurements (Ferraro & Pattanayak 2006; Pouzols et al. 2012).

Time lags are important in conservation (Maron et al. 2012). Development may result in immediate biodiversity losses, but ecological gains from compensatory conservation activities may take time to accrue. Time lags are undesirable if the existence of biodiversity provides some ongoing ecosystem service, which is diminished during the time lag (Bekessy et al. 2010). Then, even if biodiversity levels were eventually restored to predevelopment levels, the ecosystem services lost in the interim could necessitate additional compensation. Time lags may be more or less important depending upon when the intervention is evaluated (e.g., whether it is assessed 1, 10, or 100 years after the initial intervention). Although not part of the frame of reference per se, the point in time at which interventions are evaluated is critical because this often has implications for the evaluation outcome.

### Exploring Different Frames of Reference for Biodiversity Offsets

Biodiversity offsets are conservation interventions that we use as an example through which to explore the effects of reference frame specification on outcomes. We considered biodiversity offsets (henceforth, offsets) as interventions that provide additional substitution or replacement for unavoidable, negative impacts of human activity on biodiversity; involve measurable, comparable biodiversity losses and gains; and demonstrably achieve, as a minimum, no net loss (NNL) of biodiversity (Bull et al. 2013).

The use of offsets is expanding worldwide as a means to secure biodiversity alongside development activities and increase the role of the private sector in conservation activities (Gibbons & Lindenmayer 2007; Madsen et al. 2011). Offsets are useful for an exploration of reference frames because they have clearly articulated objectives: NNL or a net gain (NG) in biodiversity (McKenney & Kiesecker 2010; Madsen et al. 2011). To demonstrate NNL or NG fundamentally requires definition of a frame of reference against which to evaluate losses and gains (Gordon et al. 2011).

Our overriding objective was to explore key considerations when specifying reference frames and to determine how these affect the outcomes of conservation interventions, through the lens of biodiversity offset models. We considered an intervention successful when an offset intervention secured NNL of biodiversity. This can be measured against various reference frames, based on different biodiversity trajectories and anthropogenic impacts. We examined the implications of choosing different reference frames for interventions. We defined reference frames at the project and landscape scales and specified them as a baseline or counterfactual. We considered the impact of time lags and how the difficulty in achieving conservation success changes with different reference frames. We used 2 types of models. The first was a general (aspatial) model that allowed us to examine the best-case performance of a generalized offset policy against a set of reference frames combining 3 different biodiversity trajectories and 3 different anthropogenic impact scenarios. We also considered uncertainty in this model by assuming incomplete knowledge about the parameters governing the biodiversity trajectory. We used the second (spatial) model to test the conclusions for the combination of trajectories and impacts associated with a real example: urban development and offsetting within deteriorating grassland ecosystems around Melbourne, Australia (Gordon et al. 2011).

## Methods

### General Model

We created a generalized biodiversity offset model to explore the relationship between biodiversity trends and anthropogenic impacts when the relationship is evaluated against different frames of reference (Tables1 & 2). The model is based upon a function *B*, representing the biodiversity of some region, where *B* is a function of time *t*, and at *t* = 0, *B(t)* = *B*_0_, a constant representing initial biodiversity value. The absolute values of the model parameters are arbitrary, and those used to generate our results are in Supporting Information. The period for evaluating offset outcomes was 100 years, and the parameters were set so that all biodiversity in the region was developed or offset within this period.

**Table 2:** Key spatial and temporal factors that should be considered when specifying a frame of reference for measuring the performance of a biodiversity offset intervention.^a^

Consideration	Applies to	Example options	Explanation
Spatial scale	total area to include within the NNL calculation	project	biodiversity losses and gains compared across development and offset project sites only
		landscape	biodiversity losses and gains summed across the entire region, i.e., including the matrix of sites that are neither development nor offset
Temporal scale	offset success assessed against a current or projected level of biodiversity	fixed (baseline)	biodiversity value at some point in time measured or estimated and considered the baseline; NNL is assessed against this fixed baseline
		relative (counterfactual)	NNL is assessed against predicted future trend in biodiversity
Time lag	temporary losses in biodiversity value, between development occurring and offset maturing	include interim loss in biodiversity in baseline; do not include interim loss in biodiversity	possible to either include or exclude the summed biodiversity benefit lost due to time lags from calculations as to whether conservation objectives have been achieved
			a consideration not explicitly included in the generalized model developed here, but time lags evident in Melbourne case

a The objective of biodiversity offset interventions is no-net loss (NNL) of biodiversity overall, alongside economic development.

The quantity *B(t)* is determined by 3 functions: *dev(t)*, the amount of biodiversity lost to development over time; *off(t)*, the gain in biodiversity from offsets over time (in response to development); and *T(t)*, which describes the underlying biodiversity trajectory. In this model, the anthropogenic impacts are represented by *dev(t)* and the biodiversity trajectory by *T(t)*. In the absence of development and offsetting the biodiversity trajectory is given by(1)
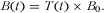
With both, the biodiversity trajectory can be written as:(2)
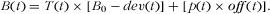
The function *p(t)* specifies how the (protected) biodiversity contained in offset locations changes over time in response to offset actions. For simplicity, we assumed the managed biodiversity within the offset remained constant (*p(t)* = 1). A more general application of this model could explore other functional forms for *p(t)* or couple *p(t)* with *T(t)*.

In the absence of any intervention, we assume biodiversity in the region can follow one of 3 trajectories: decreasing, stable, or increasing over time. The stable trajectory was modeled by a constant and the decreasing and increasing trajectories as logistic curves based upon the functional form described in Mace et al. (2008) for population decline:(3)
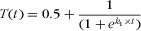
The coefficient *k*_1_ determines whether the trajectory is decreasing (positive *k*_1_) or increasing (negative *k*_1_) and its value determines the shape of the logistic function (i.e., how quickly the biodiversity component decreases or increases). These functions provide an approximate representation of biodiversity change (Mace et al. 2008). Results with other functional forms are in Supporting Information.

We assumed a linear loss of biodiversity from development over time, occurring at a constant rate determined by the parameter *k*_2_, which was negative:(4)
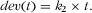
Different types of development could be modeled by substituting different functional forms into Eq. 4. Offsets associated with development were expressed as(5)
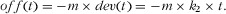
Because development impacts *dev(t)* are negative, we included a factor −1 in Eq. 5 so that offsets represented a positive gain for biodiversity. We made the optimistic assumption that offsets occur simultaneously with development and create new biodiversity immediately. There was no limit to the amount of biodiversity that could be added to the region, so development impacts could always be offset. The factor *m* in Eq. 5 multiplies the amount of biodiversity offset for a given development, meaning that if *m* = 2, twice the biodiversity lost from development would be created by the offset. Values of *m* < 1 represented the case in which offsets were only partially successful and thus created less biodiversity then development removes. Unless specified otherwise, *m* = 1 for all simulations. We also assumed that, once created, offsets are managed in perpetuity and remain of constant biodiversity no matter what form *T(t)* takes.

The parameters *B*_0_, *k*_1_, *k*_2_, and *m* and the functions *p(t)*, *T(t)*, *dev(t)*, and *off(t)* will all have uncertainties associated with them in a real ecosystem. We simulated the effect of uncertainty by varying the values of *k*_1_ and *m*; a more thorough exploration of uncertainty is beyond the scope of this study.

We generated 3 different anthropogenic impact and intervention scenarios based upon the above equations for comparison of offset performance: no development (*dev(t)* = *off(t)* = 0, so *B(t) = T(t) × B*_0_); development only (development occurs without offsetting [*m* = 0], so *B(t) = T(t) × [B_0_* − *dev(t)]*); and development with offsets (development and offsets occur, so *B(t)* is given by Eq. 2.

In combining these 3 anthropogenic impact scenarios with the 3 different biodiversity trajectories, hypothetical systems subject to different dynamics were created. We examined how *B(t)* changed over time for each anthropogenic impact scenario and biodiversity trajectory. Further details on assumptions are in Supporting Information. Although the 3 scenarios above represent the approach taken to regional biodiversity management, they can also be used as counterfactual frames of reference.

### Real World Simulation Model

The general model presented above depicts an idealistic offset process with several simplifying assumptions. To translate this into the real world, we utilized an existing model developed for biodiversity offsets associated with the clearing of native grassland to expand the city of Melbourne (Gordon et al. 2011). In our Melbourne model, we dropped the simplifying assumptions that offset gains are instantaneous and simultaneous with development, offset areas remain constant in biodiversity value, and biodiversity value can be created without limit in a landscape. Consequently, managed offset areas gradually improved rather than remaining stable, but there was a limit on the total amount by which *B(t)* could be increased.

The spatially explicit model was coded in R (R Development Core Team 2012) and developed for a region west of Melbourne. It begins with a map depicting the condition of native grassland, derived from satellite data, upon which cadastral land parcels are overlaid. The summed condition of native grassland across all parcels in the landscape is a real world equivalent of the value *B(t)* used in the general model. To model anthropogenic impacts and conservation interventions, land parcels are sequentially developed and then offsets are implemented to compensate for the resulting loss of grassland biodiversity. Offset criteria are based on rules derived from state of Victoria legislation, and require that the area multiplied by the condition of the grassland developed must result in an offset *m* times larger, such that (area × condition)_offset_ ≥ *m* × (area × condition)_developed_.

In the model of biodiversity trajectory, at each time step, the condition of native grassland evolved on different trajectories depending on current condition of the grassland and whether or not it was being managed as an offset (Supporting Information). Stochastic variation was incorporated into the model by including small random fluctuations in condition *B(t)* at each time step and by selecting parcels for offset and development randomly (but subject to constraints; see Supporting Information for details). We ran the model 25 times under each scenario and examined the average behavior of biodiversity condition *B(t)* over time. Under the Victorian scheme, “habitat hectares” (HH) are used as a metric for measuring habitat condition (Parkes et al. 2003). The quantity *B*(*t*) is therefore measured in HH condition scores summed across all land parcels in the region. But because our results are not specific to a particular biodiversity metric, we do not report HH values.

We extended the original Melbourne model in 2 ways. First, the biodiversity trajectory of unmanaged grassland was varied. In this model, a grassland biodiversity deterioration curve was derived from expert opinion and applied to all parcels of unmanaged land. We also explored the consequences of assuming the biodiversity trajectory was stable. The stable trajectory in the Melbourne case was unrealistic, but we used it to corroborate the outcomes of the general model. This was justifiable because our primary aim was not to provide a realistic assessment of the outcome of offset policies for Melbourne, but to explore the implications of the choice of reference frame. Second, the output was examined against the different frames of reference in Table2 to determine how the choice of reference frame affected offset performance.

To explore the importance of the spatial scale of the frame of reference, we noted that summing biodiversity value across all land parcels reflected evaluation against a landscape-scale frame of reference. We then assessed outcomes at a project scale, done by summing *B(t)* across only those parcels that were either offset or developed.

## Results

### General Model

**Figure 1:**
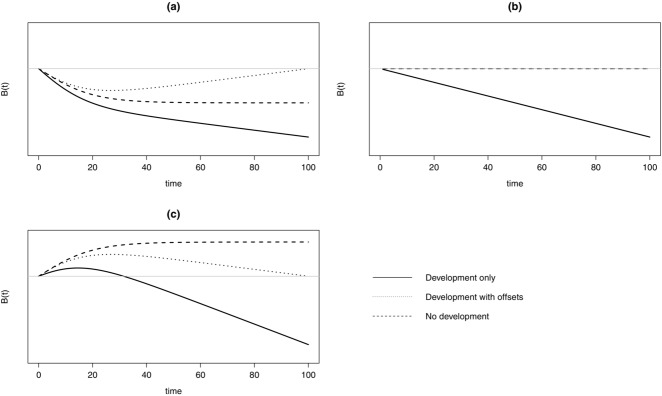
(a) Decreasing, (b) stable, and (c) increasing biodiversity (B[t]) over time under the 3 development scenarios in a general theoretical model of a hypothetical ecosystem (horizontal line through the origin represents the fixed baseline). Results were calculated at a landscape scale. No metric is specified here for biodiversity, so no scale is given, but biodiversity increases above the origin.

In all cases, against a fixed baseline, biodiversity offsetting eventually resulted in NNL of biodiversity. However, the conservation outcomes in the interim varied significantly depending on the trajectory of the ecosystem and led variously to a net loss (NL), NNL, or a NG depending on whether the biodiversity trajectory was decreasing, stable, or increasing, respectively (Fig.1). Therefore, despite that NNL was achieved after 100 years for all 3 trajectories, if the offset policy were evaluated or abandoned after 50 years, the outcomes achieved would change depending upon the biodiversity trajectory.

Under a decreasing biodiversity trajectory, the development-with-offsets scenario outperforms the no-development scenario, while this is reversed under an increasing trajectory (Table1; Figs.1a & 1c). This was a result of evaluating against a fixed baseline. When biodiversity was decreasing, development with associated offsets initially only slowed down the decline (Fig.1a). When biodiversity was already increasing, development with offsets only hampered improvement due to the loss associated with development impacts (Fig.1c). With the stable biodiversity trajectory (Fig.1b), development with offsets and no development scenarios had identical performance due to our assumption that each offset created new biodiversity equal to that what was lost to development.

**Figure 2:**
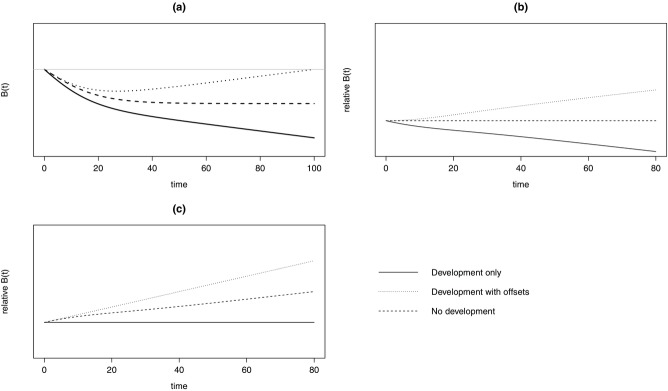
Outcomes for general model of a hypothetical ecosystem showing biodiversity (B[t]) that is on a decreasing background trajectory measured against different frames of reference as follows: (a) fixed baseline, (b) no-development counterfactual, (c) development-only counterfactual. The development-with-offsets intervention is considered a net loss of biodiversity until the 100-year mark with a fixed baseline. It is considered a net gain in biodiversity at all times for both counterfactuals (Table3).

With an NNL objective, the choice of reference frame completely determined whether and when the offset intervention was successful. In a deteriorating ecosystem (Fig.2), offsets eventually lead to NNL against the no-development or development-only counterfactuals, over the period modeled (Figs.2b & 2c). Against the fixed baseline, the development-with-offsets scenario also eventually produced NNL and outperformed the no-development counterfactual (Fig.2a). However, the fact that we assumed biodiversity remained stable rather than increased within the offset area meant that overall, biodiversity still decreased for the majority of the simulation (Fig.1; Table3).

**Table 3:** The best possible outcomes for biodiversity offset schemes under 3 different biodiversity trajectories, against both fixed and relative frames of reference.^a^

	Fixed	No	Development -
Biodiversity	current	development	only
trajectory	baseline	counterfactual	counterfactual
Decreasing	−	+	+
Stable	0	0	+
Increasing	+	−	+

a Results calculated at the landscape scale. Shown is overall biodiversity change within the first 100 years for the development plus offset scenario relative to each frame of reference. Key: −, net loss; 0, no net loss; +, net gain.

Performance against a counterfactual varied, depending on which alternative scenario was defined as the relative frame of reference. Against a no-development counterfactual, offsets improved the situation in deteriorating regions and made it worse in improving regions (Table3). Against a development-only counterfactual, offsets always led to a NG, regardless of the underlying biodiversity trajectory (Fig.2; Table3).

The results presented here were obtained assuming logistic functional forms for biodiversity trajectory. When repeated with a range of alternative forms, the results were qualitatively the same (Supporting Information).

**Figure 3:**
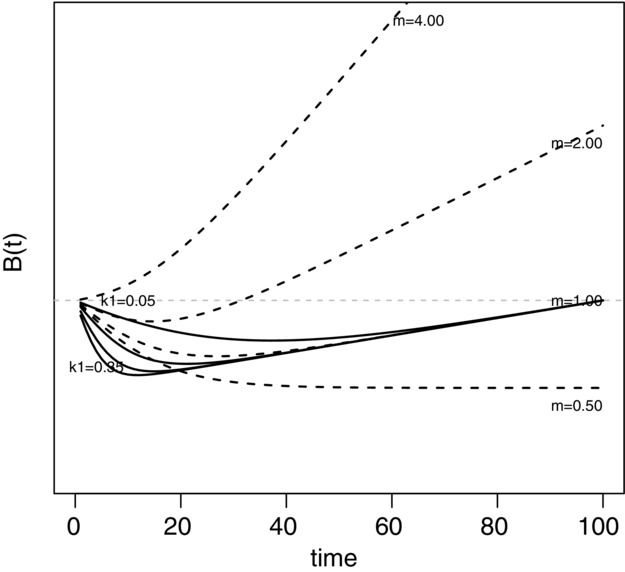
Outcomes of a development-with-offsets scenario relative to a fixed baseline of the initial level of biodiversity in the system under decreasing biodiversity for the general model of a hypothetical ecosystem. The curves show variation in parameters (see Methods for parameter definitions) used to specify the biodiversity trajectory, k_1_ (solid lines) and ratio of biodiversity value added to amount lost from the system as a result of development, m (dashed lines). When parameter m < 1 offsets are partially implemented (i.e., noncompliance); when m > 1 offset multipliers are used. No metric is specified here for biodiversity, so no scale is given, but biodiversity increases above the origin.

With decreasing biodiversity and a fixed baseline, varying *k*_1_ Eq. 3 resulted in initial variation in conservation outcomes that then converged over time (Fig.3). Conversely, varying *m* led to conservation outcomes diverging over time. This result was partly due to our implicit assumption that all biodiversity is eventually managed via offsets or lost through development, so over longer time scales the outcomes were more sensitive to offset multipliers than biodiversity trajectory. However, this may be realistic for some landscapes subject to continued development.

### Real World Simulation Model

**Figure 4:**
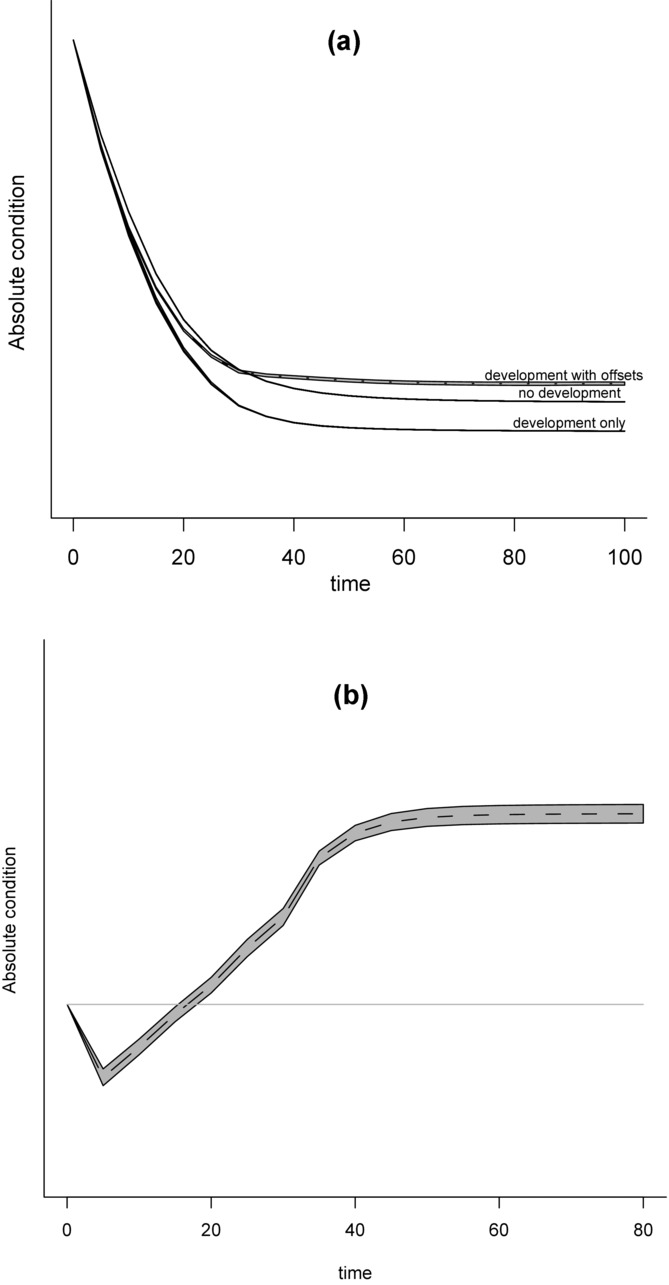
Results of simulation model of offsets for development impacts on native Melbourne grasslands: (a) summed grassland condition scores (i.e., biodiversity value) at a landscape scale for a realistically decreasing biodiversity trajectory relative to fixed baseline of overall grassland condition in the landscape at the beginning of the simulation and (b) summed grassland condition under a development-with-offsets scenario for a realistically decreasing biodiversity trajectory relative to a fixed baseline but at a project scale (i.e., representing just the development and offset sites rather than the landscape as a whole). In (b) shaded area represents variation between simulations. Both graphs show mean behavior of 25 simulations, where dashed lines represent the mean and width of each line is the standard deviation.

The Melbourne model predicted that development with offsets would result in NL against a fixed baseline, but would be an improvement upon the no-development scenario with decreasing biodiversity (Fig.4a). This is consistent with the general model (Fig.1a). However, because the Melbourne model was more realistic in including a time lag between development and the maturation of biodiversity gains in offset projects, the improvement only manifested after approximately 30 years. The length of the time lag and absolute value of NL reached over time depended on the biodiversity trajectory.

When we introduced the constraint that biodiversity within offset sites remained stable rather than increasing under management, as was the case for the general model, the Melbourne model resulted in NL against a fixed baseline. In the comparable scenario and frame of reference, the general model predicted that NNL would be achieved because of the assumed lack of time lag. These differences between the general and more realistic models emphasize that offsets may be less effective in meeting objectives once ecological limitations are considered.

Performance of offsetting at the project scale was markedly different than at the landscape scale (Fig.4b). At a project scale, because biodiversity losses occurring in the region outside of areas directly managed under the offset scheme were ignored, the offset policy resulted in a NG from approximately 20 years onward (due to time lag in offset biodiversity gains maturing), against a fixed baseline. The abrupt minimum in the curve, at approximately 5 years, was related to the point at which offsets first begin delivering gains. Specifying a counterfactual at the project scale was not possible because this frame of reference only included areas of grassland when they became either developed or managed as off sets. Thus, these areas were not defined under a no-development counterfactual, and the relative development-only curve would have been the same as the development losses curve.

## Discussion

### Measuring the Success of Conservation Interventions

The choice of reference frame for assessing performance affected the apparent success of an offset policy, even when there was no difference in absolute outcomes for biodiversity—the distinction was rather between perceived gains and losses. However, setting different frames of reference at the outset may affect how participants in the scheme behave. The choice of reference frame will affect the actions required to achieve an NNL target. Depending on how this is translated into policy, it may also affect the incentives for land managers and the degree to which they bear the cost of conservation. Land managers may carry out only the minimum restoration required for an offset, particularly if they work for commercial companies motivated by legislation rather than conservation. Against the development-only counterfactual, any minimal effort resulted in NG. But against a fixed baseline and decreasing biodiversity, achieving NNL required more biodiversity gains to be generated from offsetting than was lost from development. In this case, managers would bear the cost of providing biodiversity conservation, while society benefits from development but sacrifices no natural capital. However, against the same baseline with increasing biodiversity, a manager could provide no conservation funding and achieve NNL and society would lose an opportunity for natural capital gains.

An intuitive argument for counterfactuals is that they account for the dynamic nature of socioecological systems (Nicholson et al. 2009). However, challenges exist in setting counterfactuals, not least in terms of developing and validating a projected trend in biodiversity and anthropogenic impacts when knowledge is poor. Uncertainty is a key barrier to defining counterfactuals (TEEB 2010). Even fixed baselines are subject to measurement error and knowledge limits (Regan et al. 2002). Using a counterfactual requires strict criteria for judging predictions about trends and ongoing monitoring that continually revisits predictions to test their validity (Ferraro & Pattanayak 2006; Quétier & Lavorel 2012). These problems have been identified in relation to a range of conservation interventions such as REDD projects (Angelson 2008). If future trends are uncertain, there is also an incentive to cheat by overestimating or underestimating future biodiversity decrease to change the amount of offsetting required, depending on which stakeholder group is setting the frame of reference. As such, avoided-loss offsets (where the prevention of future, anticipated, biodiversity losses is considered a conservation gain) can make conservation practitioners, and scientists, uneasy.

### Achieving NNL against Different Frames of Reference

Against a fixed baseline at a landscape scale for a region with decreasing biodiversity, we found that ensuring NNL before the end of our simulations required multipliers. However, NG could be expected in the short term even with low or no multipliers if performance was evaluated against a no-development counterfactual. This result suggests that offsets are best equipped to meet certain NNL objectives for regions with decreasing biodiversity.

With increasing biodiversity, even best case offsets performed worse than simply preventing development, making offsets a less preferable option from a conservation perspective (Fig.1c). An implication of this is that the biodiversity trajectory and choice of reference frame could influence whether an offset policy is the best policy option for biodiversity conservation. Offsets still outperform the development-only scenario, but conservationists may not generally consider this an acceptable counterfactual.

The Melbourne model demonstrated the importance of time lags. If lags are taken into account when evaluating policy performance, then multipliers may be insufficient to ensure NNL (Moilanen et al. 2009). One option to resolve this would be to require a biodiversity banking mechanism in which offsets matured in advance of development (Bekessy et al. 2010). Our results support the idea that consideration of uncertainty is important in general for planning conservation interventions (Langford et al. 2011). Further, the fact that varying the offset multiplier *m* between 0.5 and 1.0 (where *m* < 1 suggests a proportion of offsets fail) led to divergent outcomes in the long-term suggests that offsets are highly sensitive to even low levels of noncompliance. Thus, issues around compliance might be more important then scientific knowledge about the target ecosystem, and compliance is a challenge in even the most established biodiversity offset policies (Gibbons & Lindenmayer 2007; Bull et al. 2013). This suggests that plans for ongoing monitoring and management of noncompliance should be a prerequisite for an offset policy.

### Scale of Conservation Mechanisms

Our models showed that when a frame of reference was set at a project scale, an NG is always possible, assuming there is full compliance with the offset policy, although there may be a time lag. But if offset providers claim to achieve NNL in the absence of clear definitions of scale and reference frame, stakeholders may reasonably be expecting NNL at the landscape scale. As we have intimated, offsets can only support the delivery of NNL in a deteriorating landscape with a fixed baseline at a landscape scale as part of a broader suite of conservation mechanisms.

Alternatively, a landscape scale NNL requirement may appear achievable if a regional offset policy generates substantial leakage of development outside the region (eftec 2010). That is, offsets could merely displace development activities into other regions not subject to the policy. In this case, the scheme could appear to have achieved NNL relative to even a landscape scale frame of reference, despite major development impacts having occurred elsewhere. These complexities suggest that the scale at which offsets are assessed should be carefully considered in light of the role development plays as a driver of biodiversity loss within the broader social–ecological system. Offsets could be assessed against multiple scales, although different scales may require different objectives.

The choice of whether to use a fixed or relative frame of reference, and of the spatial and temporal scale at which outcomes are evaluated, is at least partly subjective. The only crucial and unassailable requirement, from the viewpoint of conservation science, is that some kind of frame of reference should be transparently specified and the implications of the choice of frame of reference should be appreciated in advance of the intervention.

## Supporting Information

Further details on the general model (Appendix S1) and the Melbourne simulation model (Appendix S2) and the results obtained with different functional forms for biodiversity trajectory (Appendix S3) are available online. The authors are solely responsible for the content and functionality of these materials. Queries (other than absence of the material) should be directed to the corresponding author.
