# Supplementary material for: Importance of Baseline Specification in Evaluating Conservation Interventions and Achieving No Net Loss of Biodiversity
Source: Conserv Biol. 2014 Feb 13;28(3):799–809. doi: 10.1111/cobi.12243 (PMC4241037; doi:10.1111/cobi.12243)
Supplement: Supplementary file 1 — Supplementary [file cobi0028-0799-SD1.zip › BullAppendixS1.doc]

**The importance of baseline specification in evaluating conservation interventions and achieving ‘no net loss’ of biodiversity**

*Bull JW, Gordon A, Law E, Suttle KB, Milner-Gulland EJ*

**Supplementary materials 1**

The general model was entirely written in the statistical programming language R version 2.15.1 (R Development Core Team, 2012). The most important simplifying assumptions made in building the model are as follows.

*Model assumptions*

The purpose of the simple model was not to simulate real-world biodiversity offsets, but to explore the principle behind offsetting and show the effect of varying the baselines used to evaluate performance. Thus a number of unrealistic simplifying assumptions were made, ignoring the majority of design challenges to offsetting unless where explicitly stated in the manuscript.

- Biodiversity trajectory is either of constant or logistic (+ve / -ve) functional form. Alternative functional forms presented in S3
- Development ‘project’ impacts to be offset occur all at once and instantaneously
- Development impacts are all negative and linear (constant decrease in biodiversity value every time step)
- Offsets happen instantaneously and at the same time as development ‘projects’, completely negating development impacts
- Offsets are fully compliant and successfully implemented
- No limit is placed in the ability of offsetters to create extra biodiversity value equivalent to biodiversity losses, but
- Once an offset has been created it is managed, and managed land does not then follow any biodiversity trajectory
- Biodiversity losses and gains are both measurable and known exactly
- All developers and offsetters act transparently and in compliance with the offset policy
- All development is offset, nothing else except development projects and biodiversity trajectory changes overall biodiversity value

A number of these assumptions are relaxed and replaced with real-world complexity, such as time lags between development impacts and offset projects, in the predictive model (S2). An overview of some of the key design and implementation challenges in offsets can be found in Bull et al. (2013).

Model parameter values

As the model is theoretical and used to examine relationships only, the model parameters used in running simulations were arbitrary and not based on any real-world parameters. The values are consequently not included in the main text, to avoid confusion. For the sake of reproducibility, they are included in Table S1.1.

***Table S1.1***: parameter values used in theoretical model simulations

| **Parameter** | **Value** | **Range tested** |
| --- | --- | --- |
| *t* | n/a | 0 – 100 |
| *B_0_* | 500 | n/a |
| *p(t)* | 1 | n/a |
| *k_1_* | 0.1 | 0.05 – 0.35 |
| *k_2_* | -5 | n/a |
| *m* | 1 | 0.5 – 4.0 |

**References**

Bull JW, Suttle KB, Gordon A, Singh NJ, Milner-Gulland EJ (2013). Biodiversity offsets in theory and practice. Oryx doi:10.1017/S003060531200172X.

R Development Core Team (2012) R: A language and environment for statistical computing. [available at: <http://www.R-project.org/>]
