# Supplementary material for: Importance of Baseline Specification in Evaluating Conservation Interventions and Achieving No Net Loss of Biodiversity
Source: Conserv Biol. 2014 Feb 13;28(3):799–809. doi: 10.1111/cobi.12243 (PMC4241037; doi:10.1111/cobi.12243)
Supplement: Supplementary file 1 — Supplementary [file cobi0028-0799-SD1.zip › BullAppendixS2.doc]

**The importance of baseline specification in evaluating conservation interventions and achieving ‘no net loss’ of biodiversity**

*Bull JW, Gordon A, Law E, Suttle KB, Milner-Gulland EJ*

**Supplementary materials 2**

*Study area and context*

Melbourne is situated amongst some of the best remnants of Western (Basalt) Plains Natural Temperate Grassland. This vegetation community has over 99.5% of its original distribution lost or substantially altered (Williams et al., 2005) and comprises one of Australia’s most endangered ecosystems. The extent and condition of native grassland in the model is shown in Fig. S2.1.

The native grassland condition map used in this model was derived from a vegetation condition layer for the state of Victoria (GIS layer NV2005_QUAL1; DSE, 2009) and from site data collected during 2008-2009 by the Victorian Growth Areas Authority and the Victorian Government’s Department of Sustainability and Environment (DSE). Each cell represents grassland condition within a 50x50 m^2^ pixel with condition measured using the habitat hectares vegetation assessment method (Parkes et al., 2003) for areas where sites were visited and with a derived habitat hectares score from the modelled vegetation condition layer in other areas. The habitat hectares score ranges between 0-100 and represents vegetation condition relative to a mature and undisturbed benchmark of the same vegetation type. The score includes components that describe site condition (75% of score) and landscape context (25% of score). For this analysis, only the site condition component was used and the grassland condition map was then rescaled to range between 0.0 – 0.75 (Fig. S2.1).

*Grassland condition change model*

All the parameters described below were estimated based on the expert opinion of DSE ecologists. Additional references providing further background information for the condition change model are McDougall and Morgan (2005), Morgan (1998), Lunt et al. (2007) and McLaren et al. (1998).

The curves shown in Fig. S2.2 describe how future grassland condition was calculated using the *habitat hectares* metric (Parkes et al., 2003). The curves indicate that unmanaged grasslands degrade over time and that managed grasslands will improve in condition. However, once a patch of grassland falls below a certain condition, it is likely to be difficult to restore it to good condition. Thus there were managed condition curves for grassland above and below a threshold condition of 0.35 (Fig. S2.2) and when managed, grassland with a condition score above/below the threshold will asymptote towards a value of 0.75/0.35, respectively. A small proportion of pixels were allowed to cross the threshold in an upward direction due to random fluctuations and embark on the higher recoverability curve if managed. The probability of a pixel being allowed to cross the threshold was determined by expert opinion and set to 0.0005.

For each time step, the condition of each pixel of grassland was evolved using the curves in Fig. S2.2. This was done with an algorithm that operated using the following procedure on each pixel of grassland: i) The appropriate curve was chosen depending on whether the pixel of grassland was unmanaged, managed above threshold or managed below threshold; ii) The point on the curve that matched the current condition of the pixel was determined, call this *(t_1_,c_1_)*; iii) The condition of the pixel for the next time step was then determined by traversing the curve a distance determined by the time-step used in the model (call this *s*), which in this case was *s = 100* years. If the condition change curve was described by the function *f(t)*, then the new condition (*c_2_*) is given by *c_2_ = f( t_1_ + s).*

To model stochasticity of the condition change process, the condition score of each pixel was perturbed by adding a small random value sampled from a normal distribution with a mean of zero and a standard deviation of 0.02 (estimated from expert option and varied in the uncertainty analysis). Finally, moving window smoothing was applied to provide an edge effect. The edge effect models an environmental weed invasion front - where regions supporting lower condition grasslands or non-grassland supply weed propagules to adjacent comparatively weed free regions. Without active management, initially weed-free regions that are proximal to propagule sources will in turn become sources of weed propagules themselves. As such, the rate of decline in the condition of a given grassland area will depend on its spatial context at each time step. The moving window smoothing was implemented with 5x5 cell Gaussian kernel. Areas were counted as a weed source and used in the smoothing calculation if they were either non-grassland (condition score = 0) or had a condition score below 0.12 (these values were estimated from expert opinion).

**Figure S2.1** The study area west of Melbourne, Australia. The map shows grassland condition as a graduated greyscale (darker colours show higher condition) and property parcels in the study area.

**Figure S2.2** *Curves used to parameterise the grassland condition model. These curves are used to predict condition change over time for managed (solid lines) and unmanaged (dashed line) grasslands. The lower solid line represents vegetation whose initial condition is less than the 0.35 threshold value. The upper solid line represents vegetation whose initial condition is above the 0.35 threshold.*

**References**

DSE. 2009. Corporate Geospatial Data Library. Land Information Group, Department of Sustainability and Environment. Level 13, 570 Bourke Street, Melbourne, Victoria, 3000.

McDougall, K.L., and Morgan, J.W. 2005. Establishment of native grassland vegetation at Organ Pipes National Park near Melbourne, Victoria: Vegetation changes from 1989 to 2003. Ecological Management and Restoration 6:34-42.

McLaren, D.A., Stajsic, V., and Gardener, M.R. 1998. The distribution and impact of South/North American stipoid grasses (Poaceae: Stipeae) in Australia. Plant Protection Quarterly 13:62-70.

Morgan, J.W. 1998. Patterns of invasion of an urban remnant of a species-rich grassland in southeastern Australia by non-native plant species. Journal of Vegetation Science 9:181-190.

Lunt, I.D., Eldridge, D.J., Morgan J.W., and Witt, G.B. 2007. A framework to predict the effects of livestock grazing and grazing exclusion on conservation values in natural ecosystems in Australia. Australian Journal of Botany 54:401-415.

Parkes, D., Newell G., and Cheal D. 2003. Assessing the Quality of Native Vegetation: The ‘Habitat Hectares’ Approach. Ecological Management & Restoration 4:29 –38.

Williams, N.S.G., Mcdonnell, M.J., and Seager E.J. 2005. Factors in influencing the loss of an endangered ecosystem in an urbanising landscape: a case study of native grasslands from Melbourne, Australia. Landscape and Urban Planning 71:35-49.
