# Supplementary material for: Importance of Baseline Specification in Evaluating Conservation Interventions and Achieving No Net Loss of Biodiversity
Source: Conserv Biol. 2014 Feb 13;28(3):799–809. doi: 10.1111/cobi.12243 (PMC4241037; doi:10.1111/cobi.12243)
Supplement: Supplementary file 1 — Supplementary [file cobi0028-0799-SD1.zip › BullAppendixS3.doc]

**The importance of baseline specification in evaluating conservation interventions and achieving ‘no net loss’ of biodiversity**

*Bull JW, Gordon A, Law E, Suttle KB, Milner-Gulland EJ*

**Supplementary materials 3**

The functional form of biodiversity trajectory used in the main manuscript, excluding the ‘stable’ trajectory, is of logistic growth or decline. This is one of a number of possible generic trajectories for conservation targets, and so we explored the effect of using different functional forms upon model outcomes. These forms were: constant, constant proportion, logistic, and increasing proportion (Fig. S3.1), and are based upon those outlined by Mace et al. (2008).

***Figure S3.1****: different generic functional forms for biodiversity decline (reversed for biodiversity increase). Forms are (a) constant decline, (b) constant proportion decline, (c) logistic decline, and (d) increasing proportion decline. Adapted from Mace et al. (2008).*

In reality, the trajectory followed by any one conservation target might be a variant on any of these, some combination of these, or none of them. However, recent work has shown that these functional forms describe the trajectories of many Red List species very well (Di Fonzo et al., under review), and the logistic form is similar to that used by Gordon et al. (2011) for the Melbourne model (S2), and is based upon empirical measurements of grassland condition change. Consequently, we feel that for the purposes of the hypothetical model, this range of functional forms (especially when taken with their positive counterparts, and the ‘stable’ trajectory) are adequate.

When we apply the additional three non-logistic functional forms (a, b, d in Fig. S3.1) as deteriorating biodiversity trajectories in the model, and evaluate against a fixed baseline, we obtain qualitatively comparable outcomes as for the logistic form in Figure 1 of the main manuscript (Fig. S3.2, S3.3, S3.4).

***Figure S3.2****: Plots for comparison with Figure 1 in the main manuscript, but with the linear biodiversity trajectory (Fig. S3.1a). Plots of biodiversity value (‘condition’) against time under different biodiversity dynamics, using a fixed baseline (dashed grey line), on a landscape scale. No scale is given here for B(t) on the y axis, but B(t) increases away from the origin. Each plot represents a different biodiversity trajectory, with biodiversity trajectory: (a) deteriorating; (b) stable, where black and green lines coincide; and, (c) improving*

***Figure S3.3****: as for Figure S3.2, but with a constant proportional change in biodiversity trajectory (Fig. S3.1b). Note shortened time span in the x axis in (c)*

***Figure S3.4****: as for Figure S3.2, but with a increasing proportional change in biodiversity trajectory (Fig. S3.1c). Note shortened time span in the x axis in (c)*

We also repeat the analysis used to explore the influence of uncertainty in both biodiversity trajectory parameters and implementation success (*m*), via which the results in Figure 2 in the main text were obtained. Under the same conditions, but with both a linear decline and constant proportional decline in biodiversity trajectory (Fig. S3.1a, b respectively), again the outcomes are not qualitatively different and the condition curves converge (Fig. S3.5).

***Figure S3.5****: The outcomes of a ‘development with offsets’ scenario, against an absolute baseline, under two different deteriorating biodiversity condition trajectories. Subject to variation in the parameters used to specify the trajectories, as for Figure 2 in the main manuscript. Trajectory is constant (cf Fig. S3.1a) for the set of curves with minima between time = 0 and time = 20, and constant proportion (cf Fig. S3.1b) for the set of curves with minima for time > 20.*

The fact that the mode outcomes are eventually qualitatively equivalent for the range of functional forms (Fig. S3.2 – S3.4) to those found using the logistic biodiversity trajectory (Fig. 1), means that the evaluation of outcomes against counterfactuals also results in equivalent conclusions being drawn regarding the no net loss objective. Consequently, the outcomes obtained using the various different functional forms (Fig. S3.1) would result in analogous outcomes to those shown in Figure 3 in the main manuscript.

**References**

Di Fonzo, M., Collen, B., and Mace, G.M. Under review. A new method for identifying pressures acting upon wild vertebrate populations.

Gordon, A., Langford, W.T., Todd, J.A., White, M.D., and Mullerworth, D.W. 2011. Assessing the impacts of Biodiversity Offset Policies. Environmental Modeling and Software 144:558–566.

Mace, G.M., Collar, N.J., Gaston, K.J., Hilton-Taylor, C., Akcakaya, H.R., Leader-Williams, N., Milner-Gulland, E.J., and Stuart, S.N. 2008. Quantification of extinction risk: IUCN’s system for classifying threatened species. Conservation Biology 22(6):1424–1442.
